# Metabolic Health, Obesity, and Intraocular Pressure

**DOI:** 10.3390/jcm12052066

**Published:** 2023-03-06

**Authors:** Younhea Jung, Gyoung Nyun Kim, Eun Byeol Oh, Kyoung Ohn, Jung Il Moon

**Affiliations:** 1 Department of Ophthalmology, Yeouido St. Mary’s Hospital, College of Medicine, The Catholic University of Korea, Seoul 07345, Republic of Korea; 2 Health Promotion Center, Seoul St. Mary’s Hospital, College of Medicine, The Catholic University of Korea, Seoul 06591, Republic of Korea

**Keywords:** metabolic syndrome, obesity, intraocular pressure, metabolically unhealthy normal-weight, metabolically healthy obese

## Abstract

Obesity has been associated with increased intraocular pressure (IOP), but the results are inconsistent. Recently, a subgroup of obese individuals with good metabolic profiles were suggested to have better clinical outcomes than normal-weight individuals with metabolic diseases. The relationships between IOP and different combinations of obesity and metabolic health status have not been investigated. Therefore, we investigated the IOP among groups with different combinations of obesity status and metabolic health status. We examined 20,385 adults aged 19 to 85 years at the Health Promotion Center of Seoul St. Mary’s Hospital between May 2015 and April 2016. Individuals were categorized into four groups according to obesity (body mass index (BMI) ≥ 25 kg/m^2^) and metabolic health status (defined based on prior medical history or abdominal obesity, dyslipidemia, low high-density lipoprotein cholesterol, high blood pressure, or high fasting blood glucose levels upon medical examination). ANOVA and ANCOVA were performed to compare the IOP among the subgroups. The IOP of the metabolically unhealthy obese group (14.38 ± 0.06 mmHg) was the highest, followed by that of the metabolically unhealthy normal-weight group (MUNW, 14.22 ± 0.08 mmHg), then, the metabolically healthy groups (*p* < 0.001; 13.50 ± 0.05 mmHg and 13.06 ± 0.03 mmHg in the metabolically healthy obese (MHO) and metabolically healthy normal-weight groups, respectively). Subjects who were metabolically unhealthy showed higher IOP compared to their counterparts who were metabolically healthy at all BMI levels, and there was a linear increase in IOP as the number of metabolic disease components increased, but no difference between normal-weight vs. obese individuals. While obesity, metabolic health status, and each component of metabolic disease were associated with higher IOP, those who were MUNW showed higher IOP than those who were MHO, which indicates that metabolic status has a greater impact than obesity on IOP.

## 1. Introduction

Glaucoma is a progressive optic neuropathy that causes irreversible blindness, and the number of people with glaucoma is estimated to reach over 111 million in 2040 [1]. Among many risk factors, intraocular pressure (IOP) is the most important and the only modifiable risk factor for glaucoma; therefore, it is important to identify factors that affect IOP [2,3,4].

Obesity has been reported to be an independent risk factor for elevated IOP and is shown to have a positive correlation with elevated IOP in many studies [5,6,7,8,9,10]. The Beaver Dam Eye Study revealed that body mass index (BMI), the most commonly used indicator of obesity, was positively correlated with IOP [10]. In another large epidemiologic study, IOP was associated with BMI both cross-sectionally and longitudinally [7]. However, higher BMI was only marginally associated with IOP in the Barbados Eye Study [9], and another study reported no association between BMI and IOP [11].

Nevertheless, there is a growing body of literature regarding subgroups of obesity with different metabolic profiles [12,13,14]. A subgroup of obese individuals with good metabolic profiles, known as “metabolically healthy but obese (MHO)” individuals, present a favorable metabolic profile including good insulin sensitivity, a good lipid profile, and no hypertension [12]. However, metabolically unhealthy but normal-weight (MUNW) individuals, characterized by excess visceral adipose tissue deposition and adipose tissue inflammation, have been linked with serious health implications, including a higher risk of mortality, cardiovascular disease, metabolic diseases, elevated markers of systemic inflammation, and cancer [12,13,14,15,16,17].

In this context, obesity, metabolic health, and their interactions could affect the IOP. We have previously shown an association between this interaction and the onset of glaucoma; however, the possible effect of this interaction on IOP has not yet been elucidated [18].

Therefore, we sought to compare the IOP among groups with different combinations of obesity status, defined using BMI, and metabolic health status in a large cohort.

## 2. Experimental Section

This study was approved by the Institutional Review Board of the Seoul St. Mary’s Hospital, Seoul, Korea, which waived informed consent from individual subjects due to its retrospective design (KC17RESI0098). Our research adhered to the tenets of the Declaration of Helsinki.

We reviewed the data of 20,385 individuals who underwent general medical health examinations between May 2015 and April 2016 at the Health Promotion Center in Seoul St. Mary’s Hospital of the Catholic University of Korea, a 1300-bed tertiary university teaching hospital.

The medical health examination included basic laboratory tests (complete blood count and blood chemistry), urine tests, IOP measurement, anthropometric measurements, a self-questionnaire regarding previous medical history, and a chest X-ray. The examinations were performed by trained nurses, doctors, and medical laboratory technologists.

We included subjects between the ages of 19 and 85 and excluded those who were underweight or who had been previously diagnosed with glaucoma. Those who were taking systemic or ocular corticosteroids, which may affect IOP, were also excluded. Participants using eyedrops other than corticosteroids or antiglaucomatous eyedrops or systemic medications other than corticosteroids were allowed to be included in the analyses. If a subject received more than one health examination during the study period, the data from the first visit were included in the analysis.

Intraocular pressure was measured using a noncontact tonometer (Canon TX-F, Tustin, CA, USA). An average of 3 measurements for each eye were used, and the mean IOP of both eyes was used for the analyses. Anthropometric parameters and body composition values including height, weight, BMI, waist circumference, hip circumference, waist–hip ratio, skeletal muscle mass, body fat mass, and body fat percentage, were measured via a bioelectrical impedance method using Inbody 720 (Biospace, Seoul, Republic of Korea). Resting blood pressure (BP) was measured using an automatic sphygmomanometer (TM-2655P; P.M.S., Berkshire, UK) after at least five minutes of rest. Blood samples were obtained after overnight fasting from each individual’s antecubital vein and centrifuged within 30 min. Serum was analyzed for serum fasting blood glucose (FBS), triglycerides, total cholesterol, high-density lipoprotein (HDL) cholesterol, and low-density lipoprotein (LDL) cholesterol using a Hitachi 7600 autoanalyzer (Hitachi Ltd., Tokyo, Japan). The HbA1c level was analyzed using high-performance liquid chromatography (Tosoh-G8, Tosoh, Tokyo, Japan). Insulin resistance was calculated using the homeostasis model of assessment for insulin resistance (HOMA-IR) formula, as follows: fasting serum insulin (µU/mL) × FBG (mg/dL)/405 [19].

Metabolic health was determined using both the subjects’ answers to the questionnaire regarding previous medical history and health examination results. Those with three or more of the following risk factors were considered metabolically unhealthy [20,21].

1. Abdominal obesity, defined as waist circumference ≥90 cm in men or ≥ 85 cm in women for Koreans [22]
2. Dyslipidemia, defined as either previously diagnosed as dyslipidemia or triglyceride ≥150 mg/dL
3. HDL cholesterol <40 mg/dL in men or <50 mg/dL in women
4. Hypertension, defined as either previously diagnosed hypertension, systolic BP ≥ 130 mmHg, or diastolic BP ≥ 85 mmHg
5. Diabetes mellitus, defined as either previously diagnosed diabetes or FPG ≥ 100 mg/dL

Obesity phenotypes were determined based on BMI. Those with 18.5 kg/m^2^ ≤ BMI < 25 kg/m^2^ were considered of normal weight, and those with BMI ≥ 25 kg/m^2^ were considered obese, according to the revised Asia-Pacific obesity criteria [23,24].

Based on the combination of metabolic health and obesity phenotype status, the study subjects were subclassified into the following 4 groups: (i) metabolically healthy normal-weight (MHNW), defined as less than 3 metabolic risk factors and BMI < 25 kg/m^2^; (ii) metabolically healthy obese (MHO), defined as less than 3 metabolic risk factors and BMI ≥ 25 kg/m^2^; (iii) metabolically unhealthy normal-weight (MUNW), defined as 3 or more metabolic risk factors and BMI < 25 kg/m^2^; and (iv) metabolically unhealthy obese (MUO), defined as 3 or more metabolic risk factors and BMI ≥ 25 kg/m^2^.

For the statistical analyses, continuous variables were analyzed using one-way analysis of variance and categorical variables were analyzed using Pearson’s chi-squared test. In addition, an analysis of covariance was also performed adjusting for age and sex. We used the Bonferroni method to adjust the *p* values for post hoc analyses. In the sub-analysis, the mean IOP was evaluated according to the number of metabolic syndrome components in the normal-weight and obese groups. Body mass index levels were classified into 4 subgroups (normal weight: 18.5–<23 kg/m^2^, overweight: 23–<25 kg/m^2^, obese I: 25–<30 kg/m^2^, and obese II: ≥30 kg/m^2^) [24] and mean IOP was analyzed among each BMI subgroup in the metabolically healthy and unhealthy groups. For all analyses, the SPSS computer package (version 24.0; SPSS Inc., Chicago, IL, USA) was used, and a *p* value < 0.05 was considered statistically significant.

## 3. Results

**Figure 1:**
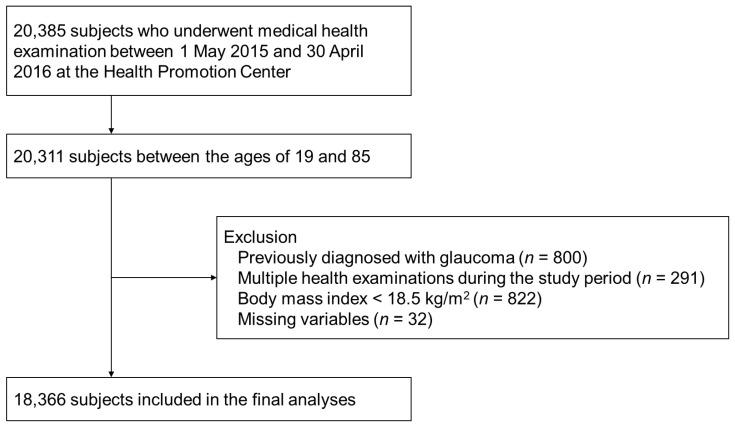
Selection of study subjects.

Figure 1 shows the selection of study subjects. A total of 20,385 subjects underwent medical health examination during the study period. In total, 18,366 subjects were included in this study. Table 1 shows the baseline characteristics of the study subjects. The number of subjects classified into the MHNW group was the largest (11,220, 61.1%), followed by MHO (3283, 17.8%), MUO (2736, 14.9%), and MUNW (1127, 6.1%). The mean age was older in the metabolically unhealthy groups (56.65 and 50.64 years in the MUNW and MUO groups, respectively) compared to metabolically health groups (46.47 and 46.57 years in the MHNW and MHO groups, respectively). The distribution of sex was different among the four groups. The mean anthropometric parameters, BMI, systolic and diastolic BP, FBG, total cholesterol, HDL, and LDL were significantly higher in the metabolically unhealthy groups compared with the metabolically healthy groups. The mean BMI values of the normal-weight groups were 22.01 kg/m^2^ and 23.23 kg/m^2^ in the MHNW and MUNW groups, respectively, whereas in the obese groups, the mean BMI values were 26.83 kg/m^2^ and 28.12 kg/m^2^ in the MHO and MAO groups, respectively, which was statistically significantly different. The metabolically unhealthy groups had a significantly higher percentage of individuals with diabetes, hypertension, and dyslipidemia.

**Figure 2:**
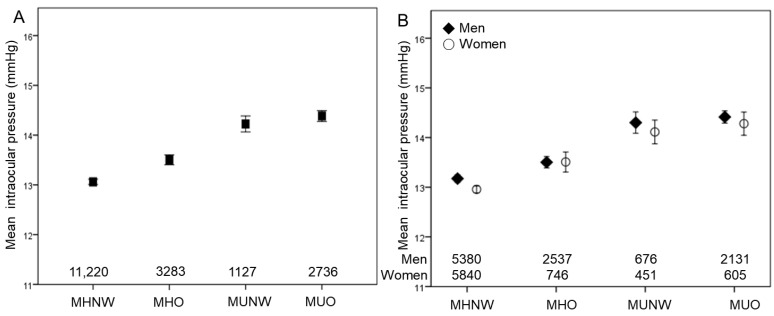
Mean intraocular pressure according to obesity and metabolic health status. Error bars indicate 95% confidence interval.

The mean IOP among the four groups showed significant differences (Table 2 and Figure 2). In the post hoc analyses, those in the MUO groups showed the highest mean IOP (14.38 mmHg), followed by those in the MUNW (14.22 mmHg) and metabolically healthy groups. The mean IOP values of the MUNW group before and after adjusting for age and sex (14.22 mmHg and 14.20 mmHg) were statistically significantly higher than the mean IOP of the MHO group (13.50 mmHg and 13.47 mmHg). The mean IOP values of the MHO (13.50 mmHg and 13.47 mmHg) and MHNW groups (13.06 mmHg and 13.08 mmHg) were not statistically significant before and after adjustment. This trend was similar in both men and women (Figure 2).

Table 2 shows the differences in mean IOP in each subgroup. Those with obesity, metabolic syndrome, or each component of metabolic syndrome showed higher mean IOP than those without. A similar pattern was observed in both men and women.

**Figure 3:**
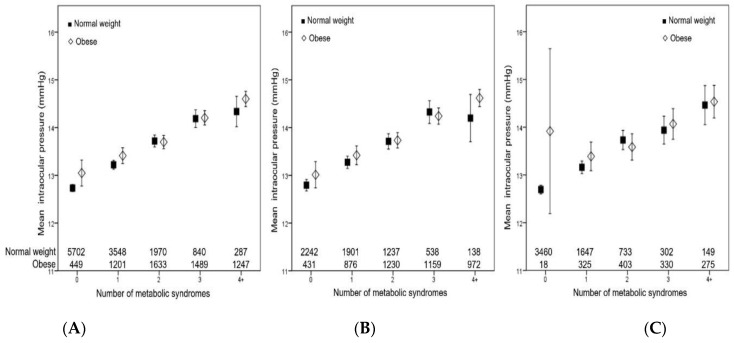
Mean intraocular pressure according to the number of metabolic syndrome components in normal-weight and obese groups (**A**) in men (**B**) and women (**C**). Error bars indicate 95% confidence interval.

**Figure 4:**
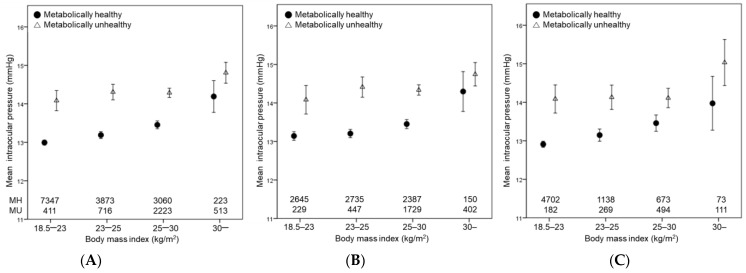
Mean intraocular pressure according to body mass index levels in metabolically healthy and unhealthy groups (**A**) in men (**B**) and women (**C**). Error bars indicate 95% confidence interval.

In the sub-analysis, the mean IOP values were compared among the subgroups according to metabolic health and BMI status. The mean IOP showed a stepwise increase as the number of metabolic syndrome components increased in both the obese and normal-weight groups in both men and women (Figure 3). There was no significant difference in subjects who were of normal weight vs. those who were obese in each group. In addition, mean IOP generally increased as the BMI level increased in both metabolically healthy and unhealthy groups in both sexes (Figure 4). Subjects who were metabolically unhealthy showed significantly higher mean IOP compared to their counterparts who were metabolically healthy in each BMI subgroup, although it was not statistically significant in the obese II group. Of note, compared to the metabolically healthy overweight and metabolically healthy obese I groups, those who were MUNW showed significantly higher IOP. These data suggest that metabolic health status is more closely associated with increased IOP than obesity.

## 4. Discussion

In this study, we analyzed a large number of subjects undergoing health examinations and compared the mean IOP after categorizing them into four groups according to obesity and metabolic status. To the best of our knowledge, this is the first study to compare IOP among groups categorized by obesity and metabolic health status. First, we found that while obesity and metabolic status are both linked with increased IOP, the MUNW group showed significantly higher IOP compared with the MHO group, indicating that metabolic health is more associated with increased IOP than obesity. This trend remained significant even after adjusting for age and sex. In addition, individuals who were metabolically unhealthy showed higher IOP compared to their counterparts who were metabolically healthy, regardless of BMI level. Additionally, there was a stepwise increase in IOP as the number of metabolic disease components increased; however, the difference was not significant between those who were of normal weight and those who were obese.

Regarding the association between obesity and elevated IOP, many previous studies have suggested a positive association. The Beaver Dam Eye Study, a population-based study performed in the USA, found that IOP was positively associated with BMI [10]. Another study examined this relationship both cross-sectionally and longitudinally in a large Japanese population and reported a positive association [7]. This positive relationship between obesity and IOP may be mediated by increased corticosteroid secretion in obese subjects and elevated episcleral venous pressure from excess orbital fat, and an increase in blood viscosity. Meanwhile, in the Barbados Eye Study, higher BMI was positively associated with IOP, but only marginally (*p* = 0.052), and another study reported similar IOP in subjects with different BMI [9,11]. Different characteristics of the study population and different study designs may explain the discrepancy between these studies. It may also be accounted for by different phenotypes of obesity. There is a large body of literature on subgroups of obesity with different metabolic profiles. Subgroups of obese individuals who are MHO have fewer components of metabolic derangements and are reported to have lower cardiometabolic risk and mortality [12,17,25,26]. On the other hand, individuals who are MUNW are characterized by excess deposition of visceral adipose tissue, inflammation of the adipose tissue, altered inflammatory profiles, and higher cardiometabolic risks [27].

In the present study, we studied the association between IOP and different combinations of obesity and metabolic health status. An important finding of our study is that the MUNW group had higher IOP than the MHO group. There could be several postulations for this finding. The MHO group had fewer components of metabolic diseases. It is well known that metabolic derangements such as hypertension, diabetes, and dyslipidemia contribute to IOP elevation [28,29,30,31]. Increased blood pressure may cause IOP elevation by increasing filtration fractions of the aqueous humor through elevated ciliary artery pressure [32]. Increased sympathetic tone and corticosteroids may also have a role in this relationship [33]. High FBG may cause IOP elevation by increasing the osmotic gradient, which can increase aqueous humor production [34]. It can also interrupt the aqueous outflow facility through fibronectin accumulation in the trabecular meshwork [35]. Autonomic dysfunction has also been proposed to be a link between diabetes and IOP [36]. Additionally, being metabolically unhealthy is related with insulin resistance, which has been reported to be associated with increased IOP, possibly through the stimulation of ocular sympathetic activity [34,37]. In our study, insulin resistance was highest in the MUO and MUNW groups, followed by the MHO and MHNW groups. Systemic inflammation may also play a role. Elevated levels of proinflammatory cytokines, including interleukin-6 and tumor necrosis factor-alpha from visceral adipose tissue, have been reported in MUNW individuals [14,38,39].

We also found that those who were metabolically unhealthy showed higher IOP compared to their counterparts who were metabolically healthy, regardless of BMI level. In addition, a stepwise increase in IOP was observed as the number of metabolic diseases increased, with no significant difference between the normal-weight vs. obese groups. These findings suggest that metabolic health status is more closely associated with IOP than obesity. The results of the present study agree with those of previous studies that have reported that each of the metabolic syndrome components was linearly and independently associated with IOP [28,29,30,31].

The strengths of our study include its large-scale design. We also used combinations of disease history as stated by each subject, as well as the results of the laboratory data, to improve the diagnostic validity of metabolic diseases. However, there are also some limitations. First, this study was conducted at a single hospital in subjects undergoing health examinations; therefore, our dataset may involve selection bias, as well as healthy user bias. In addition, information regarding underlying diseases and medications was collected from self-reported questionnaires, so our data may have included some participants using drugs than can affect IOP (i.e., corticosteroids). We tried to mitigate these potential biases by including a large number of subjects. Furthermore, this is a cross-sectional study, so we cannot determine a causal relationship. Such drawbacks warrant future clinical studies to determine the exact causal relationship between metabolic status, obesity, and IOP.

As the prevalence of obesity and metabolic syndrome is increasing worldwide, there is growing research that aims to understand the risk of diseases in subjects with MHO compared to those with MUO or MUNW. This study showed that IOP was highest in the MUO group, followed by the MUNW and MHO groups. Clinicians should be aware of the metabolic status in seemingly lean subjects, as metabolic status has a higher impact on IOP than obesity. Future studies should investigate the underlying mechanisms between metabolic health, obesity, and IOP and the therapeutic effects of lifestyle modifications and the treatment of metabolic diseases on lowering IOP.

## 5. Conclusions

In conclusion, we have shown that the MUNW group showed higher IOP compared to the MHO group. Individuals who were metabolically unhealthy showed higher IOP compared to their counterparts who were metabolically healthy, regardless of BMI level, and there was a linear increase in IOP as the number of metabolic disease components increased, but no difference between normal-weight vs. obese individuals. The present findings have potential clinical and public health implications and highlight the greater role of metabolic health status than obesity in IOP elevation.

## Figures and Tables

**Table 1:** Baseline characteristics of the study population according to obesity and metabolic health status.

	MHNW *n* = 11,220 (61.1%)	MHO *n* = 3283 (17.8%)	MUNW *n* = 1127 (6.1%)	MUO *n* = 2736 (14.9%)	*p* Value
Age (years)	46.47 ± 10.67 †‡	46.57 ± 10.08 §‖	56.65 ± 10.62 †§¶	50.64 ± 10.65 ‡‖¶	<0.001
Sex (male, %)	5380 (48.0)	2537 (77.3)	676 (60.0)	2131 (77.9)	<0.001
Height (cm)	165.95 ± 8.27 †‡	169.35 ± 8.34 *§	165.91 ± 8.79 §¶	169.21 ± 8.56 ‡¶	<0.001
Weight (kg)	60.95 ± 8.62 *†‡	77.11 ± 9.14 *§‖	64.16 ± 8.00 †§¶	80.77 ± 11.42 ‡‖¶	<0.001
Body mass index (kg/m^2^)	22.01 ± 1.71 *†‡	26.83 ± 1.89 *§‖	23.23 ± 1.33 †§¶	28.12 ± 2.62 ‡‖¶	<0.001
Waist circumference (cm)	79.09 ± 5.31 *†‡	91.37 ± 6.15 *§‖	84.78 ± 4.79 †§¶	96.26 ± 7.38 ‡‖¶	<0.001
Hip circumference (cm)	91.86 ± 4.17 *†‡	100.25 ± 4.66 *§‖	93.38 ± 4.47 †§¶	102.45 ± 5.86 ‡‖¶	<0.001
Waist–hip ratio	0.86 ± 0.04 *†‡	0.91 ± 0.04 *‖	0.91 ± 0.04 †¶	0.94 ± 0.04 ‡‖¶	<0.001
Skeletal muscle mass (kg)	24.96 ± 5.51 *†‡	31.09 ± 5.63 *§‖	25.80 ± 5.26 †§¶	31.66 ± 6.11 ‡‖¶	<0.001
Body fat mass (kg)	15.23 ± 3.31 *†‡	21.96 ± 5.01 *§‖	17.31 ± 3.37 †§¶	24.66 ± 5.81 ‡‖¶	<0.001
Body fat (%)	25.35 ± 5.70 *†‡	28.67 ± 6.16 *§‖	27.35 ± 6.13 †§¶	30.67 ± 6.00 ‡‖¶	<0.001
Systolic blood pressure (mmHg)	114.88 ± 12.79 *†‡	119.95 ± 11.44 *§‖	129.59 ± 13.45 †§	129.67 ± 12.77 ‡‖	<0.001
Diastolic blood pressure (mmHg)	71.58 ± 9.05 *†‡	75.91 ± 8.56 *§‖	78.58 ± 9.45 †§¶	80.98 ± 9.06 ‡‖¶	<0.001
Fasting blood glucose (mg/dL)	92.57 ± 13.29 *†‡	95.60 ± 13.75 *§‖	116.78 ± 32.63 †§¶	112.24 ± 27.63 ‡‖¶	<0.001
HbA1C (%)	5.49 ± 0.47 *†‡	5.56 ± 0.44 *§‖	6.19 ± 1.07 †§¶	6.04 ± 0.93 ‡‖¶	<0.001
Fasting insulin (µU/mL)	6.63 ± 2.41 *†‡	8.44 ± 3.53 *‖	8.84 ± 3.63 †¶	10.96 ± 5.16 ‡‖¶	<0.001
HOMA IR	1.58 ± 0.66 *†‡	1.98 ± 0.88 *§‖	2.76 ± 1.65 †§	3.17 ± 1.85 ‡‖	<0.001
Total cholesterol (mg/dL)	199.87 ± 34.26 *‡	204.15 ± 35.23 *§	200.49 ± 42.46 §¶	204.62 ± 40.97 ‡¶	<0.001
Triglycerides (mg/dL)	98.98 ± 62.37 *†‡	124.38 ± 71.62 *§‖	189.53 ± 119.75 †§¶	208.24 ± 136.52 ‡‖¶	<0.001
HDL (mg/dL)	58.67 ± 13.26 *†‡	51.95 ± 10.34 *§‖	47.54 ± 11.71 †§¶	45.34 ± 10.09 ‡‖¶	<0.001
LDL (mg/dL)	120.10 ± 30.72 *‡	128.91 ± 32.23 *§‖	118.20 ± 36.64 §¶	124.35 ± 35.39 ‡‖¶	<0.001
Diabetes (%)	1941 (17.3)	722 (22.0)	930 (82.5)	1991 (72.8)	<0.001
Hypertension (%)	1991 (17.7)	790 (24.1)	864 (76.7)	1949 (71.2)	<0.001
Dyslipidemia (%)	1800 (16.0)	766 (23.3)	981 (87.0)	2100 (76.8)	<0.001

Values are expressed as mean ± standard deviation or number (percentage); HbA1c = hemoglobin A1c, HDL = high-density lipoprotein cholesterol, LDL = low-density lipoprotein cholesterol, MHNW = metabolically healthy normal-weight, MHO = metabolically healthy obese, MUNW = metabolically unhealthy normal-weight, MUO = metabolically unhealthy obese; *p* values represent the results of analysis of variance or Pearson’s chi-squared test to detect differences among the four groups; the superscript symbols indicate statistically significant differences between groups with the same symbols.

**Table 2:** Mean intraocular pressure among the four groups categorized according to obesity and metabolic health status.

		MHNW (*n* = 11,220)	MHO (*n* = 3283)	MUNW (*n* = 1127)	MUO (*n* = 2736)	*p* Value	Post Hoc Analyses
Mean intraocular pressure	Model 1	13.06 ± 0.03	13.50 ± 0.05	14.22 ± 0.08	14.38 ± 0.06	<0.001	MHNW = MHO < MUNW < MUO
	Model 2	13.08 ± 0.03	13.47 ± 0.05	14.20 ± 0.09	14.34 ± 0.06	<0.001	MHNW = MHO < MUNW < MUO

Data expressed as mean ± standard error; Model 1 is non-adjusted; Model 2 is adjusted for age and sex; MHNW = metabolically healthy normal-weight, MHO = metabolically healthy obese, MUNW = metabolically unhealthy normal-weight, MUO = metabolically unhealthy obese.

## Data Availability

Due to ethical restrictions, data are available upon request from the corresponding author for researchers who get approval from the Seoul St. Mary’s Institutional Data Access Committee (http://cmccrcc.catholic.ac.kr/english/main.jsp).
